# Healthcare Staff Perspectives and Experiences of Racism’s Impact on Care for Hospitalized Black Adults: A Qualitative Study

**DOI:** 10.1007/s11606-026-10174-3

**Published:** 2026-01-29

**Authors:** Natalie Sohn, Sofia Weiss Goitiandia, Sharifa Brooks-Smith-Lowe, Catthi Ly, Lorraine M. Pereira, Amber N. Martin, Desirée Anderson, Cinnamon Rochell Etta, Trymel Harris, Lee Harrison, Linda Hills, Stephanie Morgan, Karen D. Vickers, James Washington, Sally Oh, Elizabeth Dzeng

**Affiliations:** 1 Department of Geriatrics and Palliative Medicine, Icahn School of Medicine at Mount Sinai, New York, NY USA; 2 Division of Hospital Medicine, University of California, San Francisco, San Francisco, CA USA; 3 Harvard Medical School, Boston, MA USA; 4 Community Advisory Board Member, RISE Project, San Francisco, CA USA; 5 University of California, San Francisco Benioff Children’s Hospital - Oakland, Oakland, CA USA; 6 Cicely Saunders Institute, King’s College London, London, UK

**Keywords:** Health equity, Hospital-based care, Hospital staff, ualitative methods, Racism

## Abstract

**Background:**

Racial inequities in hospital-based care for Black adults are well-documented, yet the mechanisms by which interpersonal and institutional racism contribute to these inequities remain poorly understood. Non-clinician hospital staff, such as patient care assistants, spend significant time with patients and are often members of minoritized communities, yet their perspectives on racism in the hospital are rarely studied.

**Objective:**

To investigate non-clinician staff members’ perspectives on how interpersonal and institutional racism affect hospital-based care for Black adult patients.

**Design:**

Qualitative study using in-depth, semi-structured interviews and community-based participatory research principles.

**Participants:**

Fifteen hospital staff members (patient care assistants, custodians, transporters, and ward clerks) from a large, urban, academic medical center on the West Coast. Participants self-identified as Black (27%), Hispanic (20%), and/or Asian (53%).

**Approach:**

Semi-structured interviews explored respondents’ perspectives on racism in the hospital. Interviews were analyzed using thematic analysis. A Community Advisory Board of eight Black individuals provided feedback and member checking.

**Key Results:**

Five themes emerged from the data: (1) staff members experienced workplace racism; (2) racial stereotypes appeared to contribute to inequitable care for Black patients; (3) institutional factors, such as inadequate staffing, were felt to contribute to inequitable care; (4) individual factors, such as going “above-and-beyond” job responsibilities, helped mitigate inequitable care; and (5) institutional diversity, equity, and inclusion (DEI) interventions received mixed appraisals, with staff often excluded from initiatives.

**Conclusions:**

Inequities in hospital-based care for Black patients may have roots in complex interactions between individual biases, workplace discrimination, institutional factors, and systemic underinvestment in non-clinician staff. Respondents perceived that institutional factors, such as inadequate staffing and demanding workloads, amplified racist beliefs, resulting in inequitable care for Black patients. Hospitals should invest in non-clinician hospital staff and address structural factors impeding staff members’ ability to provide equitable care to all patients.

**Supplementary Information:**

The online version contains supplementary material available at https://doi.org/10.1007/s11606-026-10174-3.

## INTRODUCTION

Decades of research have documented extensive racial inequities in hospital-based care for Black people.^1,2^ In Emergency Departments, Black patients report clinician dismissiveness, assumptions of health illiteracy, and discrimination based on perceived socioeconomic class.^3^ Black patients admitted to psychiatric hospitals are more likely to be restrained and have security services called on them than their White peers.^4,5^ Relative to White patients, Black hospitalized patients are less likely to receive adequate pain management.^6^ Inequitable hospital-based care exacerbates health disparities faced by Black patients while perpetuating health institutions’ lack of trustworthiness as safe places of healing.

**Figure 1:**
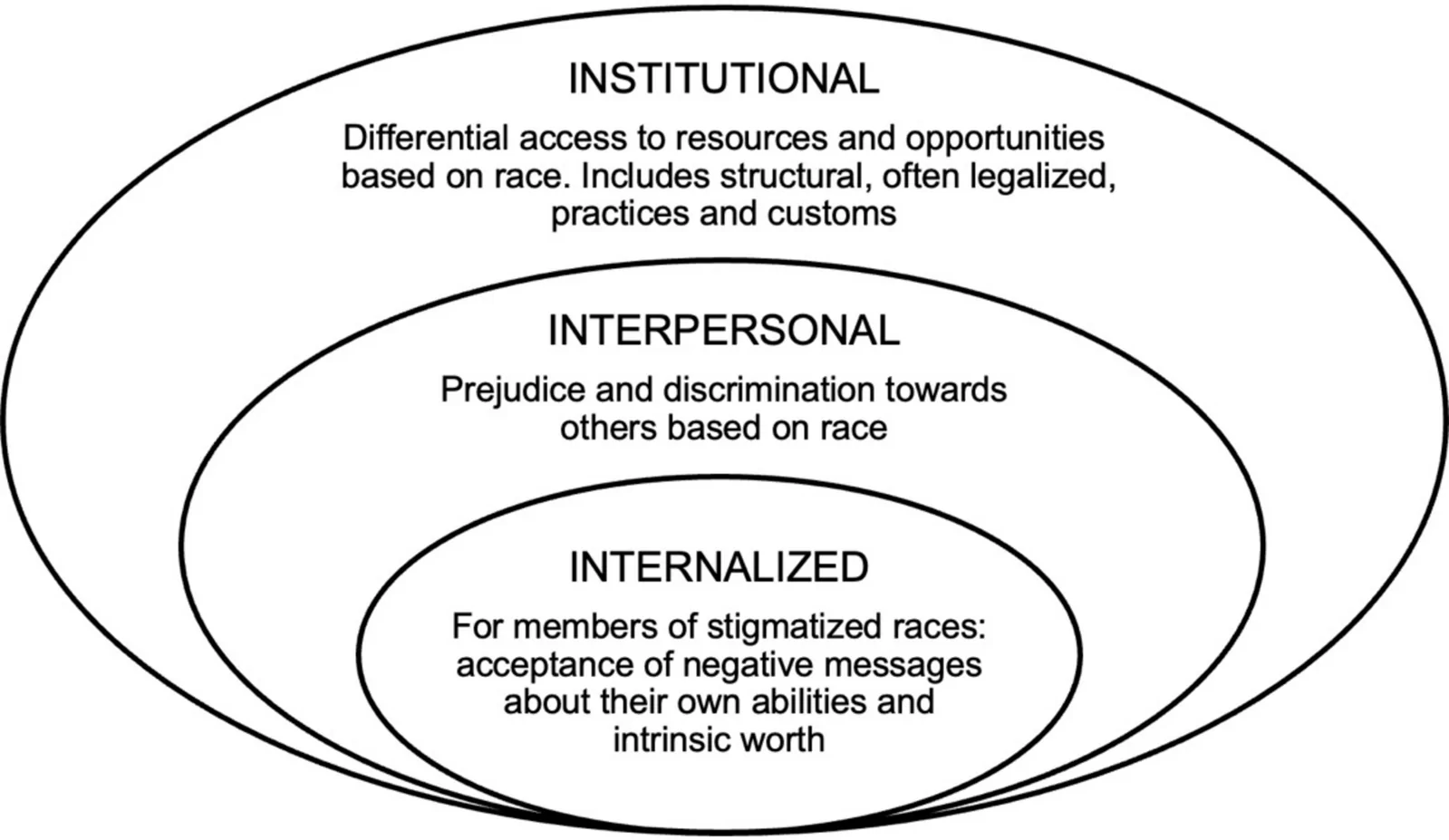
Framework for understanding racism on three levels (Jones, 2000).^5^

Though health inequities for Black individuals are well described, it is poorly understood how different levels of racism contribute to these inequities. Institutionalized racism is defined as the structural, often legalized practices and customs that lead to differential access to resources and opportunities based on race (Fig. 1).^7^ Interpersonal racism is defined as prejudice (assumptions about others’ abilities and intentions) and discrimination (differential actions towards others) based on race. Due to increased public attention to structural racism in recent years, many healthcare institutions have voiced support for health equity. Yet most institutional interventions focus on interpersonal factors, such as diversity and implicit bias training, despite limited evidence that these approaches have a lasting impact on organizational behavior change.^8,9^ Limited research has explored how institutional and systemic factors contribute to inequitable hospital-based care for Black patients.

We sought to investigate how different levels of racism affect the care of Black hospitalized adults, focusing on non-clinician hospital staff members’ perceptions of how racism impacts patients. These non-clinician hospital staff members (subsequently referred to as “hospital staff members”) include patient care assistants (PCAs) and custodial staff and are unempowered care team members compared to nurses, physicians, and other clinicians. They are typically not the targets of academic institutional investment; unlike clinicians and trainees, they rarely have access to substantive diversity, equity, and inclusion (DEI) interventions.^10^

Hospital staff members spend significant time with patients and thus have insight into their hospital experiences, while also playing a key role in care quality. Many belong to marginalized racial and ethnic groups themselves and face job-related stress from low wages and demanding work.^11^ Yet, minimal research has been conducted with this population: our literature search on qualitative interviews of mainly non-clinician hospital-based staff members in the USA yielded only a handful of articles on staff member perspectives. Some detailed experiences of burnout and insufficient health and economic protections during the COVID-19 pandemic,^12,13^ while others studied hygiene protocols,^14,15^ and one examined environmental service workers’ feelings of connectedness to clinical teams.^16^ As members of minoritized communities, many of whom are subject to inequities, their unique perspectives are crucial to understanding how various levels of racism operate in the hospital. Our objective was to elicit hospital staff members’ perspectives on how interpersonal and institutional racism affect hospital-based care for Black adult patients.

## METHODS

### Study Design

This study was conducted at a large, urban, academic medical center on the West Coast. We carried out semi-structured in-depth interviews to investigate hospital staff members’ experiences and attitudes regarding how different levels of racism affect hospital-based care for Black adult patients. Presented here is a primary data analysis of data that were collected as part of a broader study on structural racism in the hospital, for which we interviewed patients, clinicians, and non-clinical hospital staff. The medical center’s Institutional Review Board approved the study.

This project was grounded in public health critical race praxis,^17,18^ a conceptual model that guides research of complex racial phenomena and concepts. The study also incorporated community-based participatory research (CBPR), in which community members and researchers partner equitably in all aspects of the research process.^19^ Central to CBPR is the formation of a Community Advisory Board (CAB), members of which participate in study design, implementation, and dissemination. We formed a local CAB of eight Black adults, some of whom had prior experience working in healthcare. They participated in monthly focus groups with our study team to consult on various aspects of the project, including discussion of quotations, emerging themes, and manuscript review.

### Data Collection

We interviewed non-clinician hospital staff members: patient care assistants, ward clerks, custodial staff, and patient transporters. Purposive sampling of participants occurred through in-person solicitations of staff members during their hospital shifts. It also included email solicitations to staff listservs, flyers posted in break rooms, and snowball sampling. Participants provided verbal consent.

Two members of the research team recruited and interviewed participants. One interviewer (N.S.) was a resident identifying as an Asian American, White woman. The other interviewer (S.B.S.L.) was a research assistant identifying as a Black Caribbean woman. We recognize that the perceived racial, gender, and age identities of these team members, as well as power dynamics among a physician trainee, researcher, and hospital staff members, may have impacted the self-selection of staff members who participated as well as the perspectives shared. We also acknowledge our positionalities and individual backgrounds, which may have influenced data interpretation. Members of the analysis team (N.S., S.W.G., S.B.S.L., C.L., L.P., A.M., E.D.) are scholars and researchers of health equity, and included clinicians (two physicians, N.S. and E.D., a clinical mental health therapist, A.N.M.), and researchers (E.D., A.N.M., S.W.G., S.B.S.L., C.L., and L.P.). 

Interviews lasted between 30 and 90 minutes and were conducted in person or via Zoom videoconferencing. Interviews were semi-structured and open-ended, using an interview guide as a foundation while allowing participants to expand on topics of their choice. The initial interview guide was based on the public health critical race framework and literature review and evolved during subsequent interviews (Appendix A). Interviews were audiotaped, transcribed, and anonymized. Sampling occurred until theoretical saturation, the point at which interviews did not generate new themes. Interviewees received a $60 gift card.

### Data Analysis

Data collection and analysis occurred concurrently and iteratively. The themes and patterns we identified from initial interviews were incorporated into subsequent versions of the interview guide to further explore their consistency. We also compared discordant data and edge cases, analyzing how their differences impacted overarching themes. To minimize bias, we engaged in collective self-reflection on how our positionalities influenced data interpretation.

Team members (N.S., L.P., S.O., E.D.) thematically coded a subset of interviews to develop an initial codebook. Individual team members analyzed the data using thematic analysis, deductively and inductively assigning codes to portions of the text using the codebook and generating new codes when additional events, concepts, or behaviors occurred. Each code entered into the codebook included detailed explanations of assignment criteria to maximize reproducibility. Then, the team discussed codes through line-by-line analysis, building consensus on definitions and assigning codes to each line. We managed disagreements through discussion of code definitions until we achieved consensus. The rest of the research team (N.S., S.W.G., S.B.S.L., C.L., L.P., S.O.) double- and group-coded additional interviews, allowing further pattern identification and codebook refinement. A total of 40% (*n *= 6) interviews were double- or group-coded.^20,21^ Once the codebook was finalized, N.S. single-coded the remaining interviews. We conducted additional interviews to corroborate evolving hypotheses. Member checking involved presenting themes to the CAB across several meetings to cross-check interpretations for potential bias. We used ATLAS.ti software version 23.3.0 for coding.

## RESULTS

We interviewed 15 hospital staff members, including patient care assistants, custodians, transporters, and ward clerks (Table 1). Respondents self-identified as Black (27%), Hispanic (20%), Asian (53%), and/or White (7%). We generated five themes based on patterns of shared perspectives and recurring experiences.

**Table 1:** Hospital Staff Respondent Demographics

Demographic characteristic	Respondents (*n* = 15)
Role, no. of respondents (%)	
Patient care assistant	8 (53)
Custodian	3 (20)
Transporter	3 (20)
Ward clerk	1 (7)
Gender, no. of respondents (%)	
Male	6 (40)
Female	9 (60)
Race and ethnicity, self-identified, no. of respondents (%)	
White	1 (7)
Black	4 (27)
Hispanic	3 (20)
Asian	8 (53)
Age, no. of respondents (%)	
20–29	3 (20)
30–39	5 (33)
40–49	1 (7)
50–59	4 (27)
60+	2 (13)
Years worked at home institution, no. of respondents (%)	
0–4	8 (53)
5–9	4 (27)
10–14	2 (13)
15+	1 (7)

Job descriptions: patient care assistants provide direct bedside support to patients with daily activities like bathing, feeding, and mobility while assisting nurses with basic medical tasks; custodial staff maintain hospital cleanliness and infection control by cleaning patient rooms, operating areas, and common spaces according to healthcare sanitation standards; patient transporters move patients throughout the hospital using wheelchairs, stretchers, and specialized equipment for appointments, procedures, and room transfers; ward clerks handle administrative duties for hospital inpatient units including managing patient records, processing admissions and discharges, and coordinating communication between medical staff and families.

### Respondents’ Personal Experiences of Racism

Respondents recounted personal experiences of being subject to interpersonal racism within American society at large and while working within healthcare institutions. Such experiences were reported by respondents who identified as Black (Table 2, Quotation 1 (Q1)), as well as respondents identifying with other minoritized races and ethnicities. Respondents described feeling looked down upon by other employees because of the less prestigious nature of their jobs and shared stereotypes of how these jobs are often filled by people of color (Q2). One respondent shared their perspective on how cumulative racism experienced by the Black community led to generational trauma and continued to influence them and their family (Q3).

**Table 2:** Illustrative Quotations from Hospital Staff Respondents

Quotation No.	Respondent	Quotation	Self-identified race or ethnicity
Respondents’ personal experiences of racism			
1	PCA 5	“I’ve experienced racism in my life. When I was younger, I was walking to school and somebody yelled the N-word out their window.‬”‬‬	Black
2	Transporter 3	“I get treated differently because people think that I’m White…On my first week training as a transporter, a nurse [said that] I don’t look like a transporter: ‘You look like the manager, like you should be somebody.’”	Hispanic
3	Transporter 1	“A term I was taught is ‘generational trauma.’ Black people went through a lot. It makes sense that this person is acting the way they’re acting, knowing my own family history.‬ [It] is hard for them to trust. The other day I’m taking some emotional health meds to help me out and my mom is freaking out: ‘No, don’t take pills. We don’t trust that.’”‬‬‬‬‬‬‬‬‬‬‬‬‬‬‬‬‬‬‬‬‬‬‬‬‬‬‬‬‬‬‬‬‬‬‬‬‬‬‬‬‬‬‬‬‬‬‬‬‬‬‬‬‬‬‬‬‬‬‬‬‬‬	Black
4	PCA 1	“I’ve seen African American staff treated differently, coworker to coworker. [As an example,] in terms of promotions, or in [the] selection of candidates for a job position.”	Asian
5	Custodian 2	“Because you don’t speak English as well as somebody else, or you belong to a different ethnic group, you’re given harder work than other people.”	Asian
6	PCA 6	“I could tell our manager is kind of racist and [was] treating the [Black] traveler PCA differently from all of us. She was keeping an eye on her, what time she came in, what time she left… The manager [would] find the smallest thing to bring up. I felt [that] was kind of unfair.”	Asian
7	Custodian 2	“More employees are Filipino, [and], for example, [Filipino managers] give the easier room for cleaning to [Filipino employees], and a hard [room], like a COVID room, they give [to] us. The time [it takes to clean] is the same, but [there are] more risks. They hire [other Filipino staff] the easier way, they always speak Tagalog. That is discrimination.”	Asian
8	Custodian 3	“I hurt my back. [The supervisor] didn’t want to give me modified duty, [even though] everyone that doesn’t look like me [gets their] work modified. No Mexican people get it either. The Asians get it. We have no power here because our numbers are minimal. The Asians in my department, there’s so many. So, the supervisor’s afraid of them. When [nurse manager] was here, the Black lady, we had the power, they had the numbers, but they got her out of here.”	Black
9	Custodian 2	“Nobody listens [to us] because all our coworkers are very low education, and they don’t know how to protect our right[s]. Whatever they give [us as an] assignment, we have to do, because we need jobs. When I complain, they give punishment to us.”	Asian
10	PCA 7	“A White patient that called me the N-word a few times. [In that situation] I’ll say, ‘I’ll let you calm down. I’ll give you five or ten minutes. I’ll come back.’ Sometimes, that’ll work.”	Black
11	PCA 3	“The doctor was Asian, and [the patient] was so aggressive to him. He was telling him, ‘You don’t know anything about this country. This is our country. Go back to your country.’”	Asian
Racial stereotypes and inequities in patient care			
12	PCA 6	“Some caregivers get more annoyed and less patient with the older Black patients compared to Asian patients. I catch myself doing that also. It’s not fair, but sometimes that’s something I can’t control. Older Black people either have a lot of attitude or they’re really quiet. So, you have to pry [to find out] what they want. It’s more work for us.”	Asian
13	PCA 3	“You always feel [Black patients] have something in their hand or something they’re going to pull out and hit you with. You get scared. There are also White people with a lot of issues that make nurses and other staff scared. But comparing an African American patient and a White patient, the African American patients are more aggressive.”	Asian
14	Transporter 2	“What I notice is [Black patients] are always asking for strong medicine, and they’re always asking to get more and more. It’s called Dilaudid. They’re like, ‘I want it right now.’ The other patients, like a White older man, they wait for the [next dose’s] time. And Black [patients], they want it right away and they get frustrated. I think they’re more impatient.”	Hispanic
15	Custodian 1	“The receptionists were really being racist and unfair to an older lady [who] came in for an emergency. I believe it was because she was a lady, she was African American, and lower class because of her [Medicaid] insurance.”	Hispanic
16	PCA 6	“We tend to have pretty bulky Black patients, and as caregivers we see that as a lot of work. We have to do more for them, especially cleaning. And sometimes they smell [like] cigarettes or drugs like weed. That also affects how we want to take care of them. Sometimes coworkers make small remarks of how the patient looks and [they are] grimacing.”	Asian
17	Custodian 1	“It wasn’t fair for her to sit in her feces and not be tended to. I really felt that it was because she was [a patient] of color. Other people were getting the full treatment they needed who weren’t half as sick as she was.”	Hispanic
18	PCA 6	“I notice a lot of my coworkers [are] kind of racist. A lot of them refuse to go into the rooms. Sometimes they switch patients with me. Sometimes one insult from a Black older patient scares them away compared to if they got verbally abused by someone their same race.”	Asian
19	PCA 6	“For an Asian or a White patient that [is] disruptive or always yelling, they have to escalate it to the top for us to call security. But for Black patients, they don’t really try to de-escalate the situation. They just automatically bring up security to scare them like, ‘Oh, we’re calling security up here. You better get your act right.’ They try to discipline them compared to waiting [to see] if they calm down.”	Asian
20	Transporter 3	“I took down a [Black] patient; he had an infiltrated IV and it is very painful. The tech, a Caucasian man, is getting frustrated with the complaining…They have an altercation. I’m like, ‘You’re aggravating this patient.’ Are we on the verge of…Calling security? A patient is experiencing pain that he’s never felt before…You [have to] realize, ‘Of course you’re going to be frustrated.’…Some people call [security] prematurely…There are words you can use to calm the patient down, or you can keep proving your point and make the patient aggravated. I’ve seen patients almost get gaslighted into them calling [security]…Like ‘[I] need you to calm down. If you’re going to be so hostile, I’m going to have to call security,’ when the patient’s relaxed.”‬‬	Hispanic
Institutional factors contributed to perceptions of inequitable care			
21	Custodian 3	“I work two jobs because [hospital] don’t pay enough. I make $30 an hour. It’s not enough to survive here. I work my other job from 5:00 AM to 1:00 PM and then I come to the hospital parking lot and sleep until 2:30 PM. And then I go to work [at the hospital] from 3:00 PM to 11:30 PM. I sleep in my car from 11:30 PM to 4:30 AM. Then I make my way to [my other job]. If I commute home, gas costs me $800 a month. I’m not going to spend that. So, I stay. I work six days. There are a lot of people I know that do this.”	Black
22	Transporter 3	“When you go to the strikes, you see it is mostly people of color in line. We’ve done two strikes in the last five months, and they’re asking us to do more. I want the wages to go up…It seems like [the low pay] is a bit unfair.”	Hispanic
23	Transporter 1	“Our department is super busy. It’s nonstop on your feet. I’m exhausted and I have limits and it’s just back-to-back-to-back. I hear and see [people feel] exhausted, don’t feel appreciated from the department. There’s not any meaningful change happening. You keep raising the alarm saying ‘We’re exhausted, we need more people or less patients.’”	Black
24	Transporter 3	“Nurses and techs are so overworked…That is where the frustration builds up. It’s too much workflow and not enough resources, transporters, CT techs, and scanners. Everyone wants to pump it out…The dispatchers are assigning you the next job. If you are waiting too long with the patient, they’re very short with us. ‘They’re not ready? Okay. Cancel.’…I advocate for the patient, that they need to go and get their scan…There’s a production list of how many transports every transporter does in a month. They’re looking at us as numbers…We are pressed less about our compassion on the job than we are about our production…I get that there is a workflow, but we would hear less negative experiences [from patients] if our staff was trained to be more compassionate than, ‘You let them sit in a dirty bed, you didn’t tell the nurse anything, but you’re on your next transport, so good job!’”	Hispanic
25	PCA 4	“Nursing [has] become a business industry. Hospital is a business industry. We’re all overworked and sometimes we have to take a step back and have that compassion, see the people who they are regardless of their color.”	Asian
26	PCA 3	“Sometimes, especially when I’m in [the] ED, I see [older Black] patients in the hallway, and nobody comes [to] help them. [They’re] asking for help, but people keep walking by, and nobody wants to help them. The unit is so busy; they need more staff. The patient is waiting and waiting and asking for so long and nobody helps them.”	Asian
27	PCA 1	“I don’t see it [as] negligence; it’s just that there’s not much care. You’ll notice that when the [patients are] African American, the care is not as much. [There is] less total time that people are spending on care for elderly patients, especially elderly African American patients.”	Asian
28	PCA 4	“It’s hard to say that it’s intentional because [it’s] a busy hospital. Everyone is overlooked, overworked, burnt out. So, at times when you care for African American patients, you have to take a step back and give all your attention…Because they have certain expectations because of the trauma they have been through. They may think you are not giving equitable care because there are needs that need to be met.”	Asian
29	PCA 7	“COVID-positive patients get the paper tray. The wife said, ‘We’re not slaves, we’re not going to eat off a paper tray. We want the regular tray. They don’t treat White people like that; they’re just doing this because we’re Black.’ I did tell her ‘When people have COVID, they get the disposable tray, so they can just throw it away.’ She said, ‘I didn’t see it like that. They didn’t explain it like that.’ But this is how it normally works…They were complaining that they were in the [emergency department] for three days. Well, they had to wait until a bed [opens]. That’s how it normally works.”	Black
30	Transporter 3	“There are times where I think the [patient] might be thinking I’m not doing the right thing, or I’m being insensitive, but I’m just going about my job. For example, before you pick up someone’s things, you put gloves on. Sometimes patients or their family members think I’m doing it because they’re a certain race, [and] I don’t want to touch their things. But I am doing it because that’s my job and I’m doing it to be safe; I’m in a hospital. You just got to explain the protocols.”	Hispanic
31	PCA 8	“In our unit, PCAs are at the bottom of the totem pole. There is a subconscious ladder, I would say. For that reason, I was kind of hesitant on speaking up.”	Asian
32	Custodian 1	“I [called] the nurse. I told her [this patient] has to use the bathroom. But they told me that because I wasn’t a PCA, I wasn’t able to [help her] do that. My supervisor told me, ‘I understand you want to help, but that is not our job.’ I said, ‘Okay, but it’s this person’s job and she’s not doing it. Where is the justice with that?’”	Hispanic
33	PCA 8	“We do have a lot of situations where people speak out against treatment of other staff members. But our management is not the most supportive. So sometimes people hesitate to seek out [help]. None of the management really care about you, they don’t care about your problems, they just want you to work.”	Asian
Individual factors appeared to mitigate inequitable care			
34	PCA 5	“Sometimes African American patients do better with African American nurses and PCAs because they feel more comfortable. They feel like they’re being listened to, they’re being heard. It’s someone of their kind. A lot of times that’s how I’ll defuse situations. [I will] sit there and try to listen, try to comfort them.”	Black
35	Custodian 2	“If there is a White patient and White staff, they treat properly. And when there is White [staff] and Black patient, they do not treat properly. Most of the RNs and PCAs are in the Tagalog group, and when there is a Tagalog patient, we have to [do] more unnecessary care, unnecessary cleaning the room.”	Asian
36	PCA 6	“Sometimes if they’re an older Asian person and their nurse is Asian, they would be more empathetic. Compared to an Asian nurse taking care of an older Black person, the care is routine but is not as in depth. An example is maybe an Asian nurse would offer an Asian older patient other ways to be able to deal with pain, offering ice packs, hot packs, and then sometimes if they go deal with a Black older person, they would just find another pain medication that they can drink.”	Asian
37	PCA 3	“[If] I have any African American patients in ICU, I always think he’s my family, and that’s how I treat [them]. And that’s how it’s supposed to be. You have to treat your patient like your own family, your parents or grandparents.”	Asian
38	Clerk 1	“I think what about [if] I was in their shoes. Eventually, I’ll become a patient. So, I’m trying to be patient.”	Asian
39	Transporter 1	“That’s how I help patients. I’m trying to make it a normal thing for them because it is normal for people to be sick. On my end, I’m trying to make them feel a lot more comfortable and safer being in the hospital.”	Black
40	PCA 4	“[If] a patient wants me to do something that may or may not be appropriate [to my job description], I still have to meet their expectation. Especially when it comes to hygiene stuff, sometimes you are asked to do something that may or may not be appropriate, but it is what they do for their hygiene. [I] have to follow and meet their expectation. You want to make sure they’re getting the care they need.”	Asian
41	PCA 6	“[Whether] Black or Asian, we always have those patients that are needy. But it depends on how [the staff] approaches the situation. I don’t know if there’s any other way to resolve that problem besides just being able to hear out what the patient wants to do. Just communicate with them, trying to negotiate how to do it without them getting mad and without us getting irritated. Communicate your perspective and then hear what they need to do and then come to a[n] agreement on what’s the best way to move forward.”	Asian
Efficacy of institutional-level interventions in promoting equitable care			
42	PCA 5	“As far as [hospital system], they go by their terms and their policy, and they do not do racism, they do not do sexism. And when they say that, they mean it.”	Black
43	PCA 1	“I love the way [hospital system] is into [DEI]. [It’s] very diverse, and acknowledges different races and cultural practices, so that makes you feel you belong.”	Asian
44	Custodian 3	“[When] the Black Lives Matter movement first started, there was a big meeting and three White men, the CFO and CEOs, were [saying], ‘We know how the Black people were being treated here. We know you guys have problems here and it has to stop. And Black Lives Matter.’ I thought he was trying to cover his own ass…They didn’t want no riot or problems here. And it could happen. We could have protests. These White men are the ones who are in charge and get all the money. And they didn’t want anything to disrupt their money. It was just something that all so-called liberal White people were doing at that time.”	Black
45	PCA 1	“Since I joined DEI [Council], I’m more conscious about what it means to give good care to different races, especially the elderly. We are learning to be strong advocates for it in terms of patient care, to have a good, healthy unit that fosters diversity, inclusion, ethnicity, and equality…I [also] had this DEI champion training, and I was the only PCA and all of them were doctors. It was a great experience because…in my position, I don’t really get to talk to doctors.”	Asian
46	PCA 8	“At some point we did have a DEI council, [but] it doesn’t really exist anymore. I wish we did have that DEI council. I feel like that really would’ve made a difference. I do feel like that was fizzled out because of management.”	Asian
47	PCA 8	“It’s an annual thing, so we keep having to take these online classes. One of them is specifically about diversity. I’m pretty sure a lot of people just get through those things, don’t really focus on or internalize whatever they’re teaching.”	Asian
48	PCA 2	“[The online training] really helps. Then if you experience it [unjust situation], you would be able to tell somebody right away that this is happening right now and should be stopped.”	Asian
49	PCA 2	“I’ve heard [a case of] discriminating [against] an employee just because they’re new or just because they’re physically different, like they’re short or they’re big, and calling them names, making fun about it. They told somebody. They even went to the hotline [but] nothing happened. They’re not even investigating what happened.”	Asian

Further, respondents highlighted their observations of racism in workplace practices, such as hiring (Q4), task allocation (Q5), and performance assessment (Q6). These incidents were reported as firsthand experiences (Q5) and situations affecting colleagues (Q4, Q6). Respondents again noted that this racism impacted individuals from multiple minoritized racial and ethnic backgrounds. It was also perceived to intersect with English language proficiency (Q5).

Respondents situated their descriptions of workplace racial discrimination in the context of tensions between employees from different racial and ethnic groups. This appeared to contribute to “in-group” and “out-group” workplace dynamics, especially those perceived to exist between Asian and non-Asian staff (Q7, Q8). One respondent reflected upon these tensions as related to feelings of disempowerment among staff members in advocating for themselves (Q9).

In addition to describing racism perpetrated by other employees, some respondents experienced or observed racism by patients directed toward employees. Multiple respondents described being called racial slurs or insulted because of their race (Q10, Q11).

### Racial Stereotypes and Inequities in Patient Care

Respondents described instances in which they or other staff members perpetuated racist stereotypes against Black patients. Respondents also felt that hospitalized Black patients received worse care compared to non-Black patients. One racist stereotype noted was that Black patients were more likely to be “difficult” and challenging to care for than non-Black patients (Q12). Another recurring stereotype was that Black patients were more aggressive. Multiple staff members endorsed feeling afraid of Black patients and viewing them as a threat (Q13). One respondent shared a belief that Black patients were more likely to seek pain medications (Q14).

Racial stereotypes were expressed in conjunction with biases around other intersectional aspects of patient identity, including socioeconomic status (Q15), gender (Q15), age (Q12, Q27), substance use (Q16), and body size (Q16). Respondents described how perceptions of these identities exacerbated racist stereotyping, although respondents displayed varying levels of awareness of these stereotypes. Some appeared unaware of their explicit biases (Q13, Q14), while others recognized their biases and how they influenced their actions (Q12). Instances of racist stereotyping and discrimination seemed to affect staff members’ relationships with patients, resulting in more adversarial dynamics (Q12, Q13, Q16).

A recurrent observation was that Black patients’ nursing and personal care were of poorer quality and more infrequently administered than that of non-Black patients. Respondents perceived that Black patients’ care was delayed and rushed (Q17). Some felt that PCAs and nurses spent less time with Black patients and were more likely to avoid going into their rooms, especially if patients were older. Respondents noted that staff were less tolerant of difficult interactions with Black patients, leading to avoidance of caregiving duties (Q18).

In addition to inequities in care quality, some respondents noted differences in how hospital security services were deployed in response to Black compared to non-Black patients. While some felt that there were no racial differences in how security was dispatched, others believed that Black patients were sometimes unfairly labeled as threatening (Q13, Q18), resulting in a lower threshold to involve security services (Q19). Some described how, rather than attempting de-escalation and finding ways to cope with behavior that staff perceived as disruptive, security was called too early and wielded as a disciplinary tool against Black patients (Q19, Q20).

### Institutional Factors Contributed to Perceptions of Inequitable Care

Participants described several modifying institutional-level factors that affected their perceptions of quality of care. Multiple respondents described challenging working conditions for staff members in lower-paying jobs. Some shared that they worked multiple jobs (Q21) or had to go on strike (Q22) in hopes of better wages. They described their demanding, physically exhausting jobs and management’s expectations of high work output (Q23). Some perceived that this focus on high turnover and work output was related to their inability to provide compassionate care, led to frustration with coworkers and patients, and caused burnout (Q24).

Respondents viewed the hospital as operating like a business, driven by profit to the detriment of equitable care (Q25). Indeed, participants described how a lack of institutional resources, such as adequate staffing, sometimes meant that Black patients disproportionately received suboptimal care. Participants mentioned feeling that when staff experienced constraints on how much time they could spend with patients, this more significantly affected Black patients (Q26, Q27). Respondents expressed uncertainty over whether such actions by staff were intentionally discriminatory. Nonetheless, some agreed it could perpetuate mistreatment against Black patients and be perceived by patients as discriminatory, especially given the racial trauma that Black patients may have experienced (Q28).

In addition to inadequate staffing levels, respondents reported feeling that strict institutional policies sometimes conflicted with individual patient needs. Though hospital regulations applied to patients of all races, respondents noted instances when Black patients and caregivers perceived racist undertones to these rules, feeling they had received discriminatory, poorer quality treatment (Q29, Q30). Tensions worsened in cases when hospital protocol was not explained fully to patients due to a lack of staff members’ available time, or of protocols and resources for providing clarity to patients (Q29, Q30).

Another institutional factor reported to hinder equitable care was hierarchy among hospital roles, wherein clinicians (i.e., doctors and nurses) and non-clinical supervisors held more power than staff. Some respondents found it challenging for team members to feel empowered to raise concerns and advocate for Black patients within the typical hierarchical structures of clinical teams (Q31). Despite staff members’ efforts to advocate for patients or colleagues, they felt there were times they could not change an unjust situation because their superior decided not to act or was not invested in acting out of step with the status quo (Q32, Q33).

### Individual Factors Appeared to Mitigate Inequitable Care

Various individual-level factors pertaining to both staff members and patients were reported to affect the quality of hospital-based care for Black patients. Respondents discussed the positive effects of shared backgrounds between patients and staff. Many pointed to racial concordance as helping build rapport and trust (Q34). Some participants felt patient-staff racial discordance was associated with poorer care quality (Q35, Q36).

Staff members also reported that individual qualities helped them provide higher-quality, equitable care. Respondents shared how empathy for patients drove them to provide compassionate care for all patients (Q37, Q38). This illustrates how recognizing common identities beyond race, such as seeing the patient as a family member (Q37), fostered positive patient-staff relationships. Others described a strong sense of purpose in their work to normalize the experience of being ill and provide an environment of comfort and safety (Q39).

When staff members went above and beyond their job descriptions, spending extra time to establish rapport and solve problems, they thought it helped patients feel they had received adequate care (Q40). In situations where patients felt their needs were not met, respondents described negotiating with them to improve therapeutic relationships (Q41).

### Efficacy of Institutional-Level Interventions in Promoting Equitable Care

Respondents expressed mixed views toward institutional-level actions, including the health system’s stated values, specific DEI initiatives, mandatory trainings, and their effects on patient care and workplace quality. Some mentioned how the institution’s stated values of promoting diversity and equity fostered a workplace in which staff felt valued, and discrimination was deemed unacceptable (Q42, Q43). At the broadest level, such efforts were described in terms of the institution having anti-racist values, which were perceived by some to translate into an anti-racist culture. Others felt that despite stated values and displays of solidarity, these were grounded in ulterior motives of avoiding protests and interruptions to routine care (Q44). Some respondents appreciated pro-DEI language and supportive statements, but felt the institution did not support its espoused values with action to improve the workplace.

Other examples of efforts proposed to mitigate anti-Black racism included DEI councils, which were established for select hospital units and involved voluntary education and training on DEI-related topics. Some felt this contributed to improved patient care and fostered advocacy skills, especially for older adults of different races, and created a more inclusive environment (Q45). However, respondents recognized that such institutional initiatives were not usually oriented toward hospital staff, but toward clinical roles (Q45). Some also noted that these councils had been disbanded before they had a chance to participate (Q46).

Regarding employees’ mandatory computer modules about diversity, some felt they had minimal impact on behavior change (Q47), while others thought they taught staff to advocate for patients (Q48). One employee mentioned a hotline, which was meant to be a resource for staff experiencing discrimination, but felt it was not effective (Q49).

## DISCUSSION

This study elicited staff members’ perspectives regarding racism’s effects on patient care and identified institutional barriers to equitable care. Hospital staff members’ perspectives have previously been underrepresented in the scientific literature and in discussions of anti-racism in healthcare. Our findings reframe racial inequities in hospital care as products of not just interpersonal racism, but also institutional and systemic factors.

To describe healthcare inequities for Black patients, clinical literature often focuses on discrimination in clinical care, such as inequities in pain management^6^ or in the treatment of acute myocardial infarctions.^22^ We add to this body of work by describing how hospital staff members perceive racism’s effects on inequities in direct, non-clinical patient care. Respondents shed light on both the interpersonal dynamics involving deeply ingrained racist stereotypes about Black patients as “aggressive” (Q13) and the underlying institutional factors of high-stress environments and inadequate staffing levels (Q24) that seem to catalyze the increased deployment of security services for Black patients^4,5,23^ instead of de-escalation attempts (Q19). Staff members alluded to a possible cascade effect of these relationships: how the strain of their underpaid, demanding roles might lead to decreased frustration tolerances toward and poorer communication with Black patients (Q12), leading to patient frustration (Q29), resulting in staff applying racist stereotypes (Q18), which could result in actual or perceived inequitable care.

Some staff members shared how they averted conflict by spending extra time with patients to explain protocols, listen to frustrations, and negotiate compromises (Q41). Yet going “above-and-beyond” their role was reported to take time and energy, often in short supply. Further, this care was perceived not to be recognized by the institution, which prioritized rapid completion of tasks over care quality (Q24). Studies on the relationship between patient volume and nurse staffing levels have shown that higher patient-nurse ratios lead to less time spent on patient care and an increased likelihood of negative staff-patient interactions.^24^ This study suggests similar consequences for staff members with an important nuance: when there was insufficient time or energy to provide patient-centered care, some respondents felt that Black patients’ care suffered more (Q26–27). The takeaway should not be that non-clinician staff members hold more racist views than clinicians; there is no evidence that they are more likely to hold these stereotypes. Rather, it appears that racist beliefs reflecting entrenched societal stereotypes and institutional barriers, such as high staff workloads, may interact to result in discriminatory hospital care.

Our data highlight how past lived experiences with racism might affect patient-staff interpersonal dynamics. Nearly all minoritized staff members had experienced racism, including in hiring practices (Q4), workload distribution (Q5–8), and staff hierarchy (Q2). Some felt powerless and as if they were not valued as individuals (Q9, Q23, Q33). Yet some of these same staff members who had been subjected to racism appeared to hold racist stereotypes about Black people and described ways these beliefs impacted their patient care. This phenomenon aligns with the theory of lateral violence, which describes how members of oppressed groups may redirect negative behaviors toward other marginalized individuals rather than challenging the dominant power structure.^25,26^ In healthcare, this may manifest when staff who experience discrimination perpetuate similar treatment toward patients from other marginalized groups. Some respondents expressed views that configured racial groups as monolithic entities with predetermined characteristics, rather than as socially constructed categories comprising diverse individuals. Perhaps being treated as a person from a monolithic racial group perpetuated the idea that races do indeed exist beyond purely a social invention, thus justifying racial stereotypes against Black patients. This illustrates the concept of internalized oppression, which describes how individuals who have repeatedly experienced racist treatment may unconsciously absorb these underlying beliefs, then unwittingly express them to others.^27^ Implicit bias formation describes a similar concept in which repeated exposure to racist treatment can strengthen unconscious racist stereotypes.^28^ Thus, the way staff members treat others could have been based on how they were treated in the past. We theorize that this cyclical process may have played a role in how staff members’ personal experiences of racism shape racial care inequities.

Our results further shed light on hospital staff’s working conditions and suggest they are crucial to building therapeutic relationships and delivering high-quality care. These staff members are often people of color and members of minoritized communities,^29^ as evidenced by the demographics of our respondents, none of whom identified as White and non-Hispanic. Indeed, data from staff members’ home institution reveal that they are more likely to belong to racial and ethnic minorities than faculty clinicians: 31.4% of staff members identify as White compared to 56.5% of faculty clinicians; 39.8% identify as Asian compared to 28.4% of faculty; 14.9% identify as Hispanic compared to 6.3% of faculty; and 7.8% identify as Black compared to 3.9% of faculty.^30^ The importance of these non-clinician, frontline staff members’ work was highlighted during the COVID-19 pandemic,^12,13^ yet they continue to experience low wages ($13.48/hour on average),^31^ high rates of work-related injury,^32^ and poor job mobility.^33^ Respondents described how low wages made it difficult to live in the expensive neighborhoods surrounding the hospital (Q21). This led to long commutes, working multiple jobs, and needing to participate in organized strikes for higher wages. Furthermore, our findings suggest that staff members bear the brunt of inadequate staffing levels, resulting in demanding workloads such as rapid turnover of patient rooms without breaks and insufficient PCAs to respond to patient needs. The least well-compensated, most unempowered members of the care team, who can have an outsized influence on patients’ hospital experience, operate under challenging conditions.

Hospitals employ standardized protocols and policies to govern care. Patient experiences of the hospital are influenced by these uniform rules. However, “race-neutral” policies are not experienced as neutral. Standardized policies can have drastically different effects on people from diverse backgrounds, perpetuating racism and causing patients to perceive racism in their care. For example, one respondent shared how a patient’s wife had perceived paper food trays, which were given to all patients infected with COVID-19, as an act of racism, as if her husband was being treated as a slave (Q29). This illustrates how a “race-neutral” institutional policy may still be regarded as racist by patients whose experiences over the life course have been marked by inequitable treatment. Another respondent described how an older Black woman who had to wait longer than other patients for an urgent dental issue was perceived to be treated unfairly and given delayed care due to her race and low socioeconomic status (Q15). Given the secondhand nature of these interviews, we cannot know for certain whether this instance represented a case of true inequity, although there is data demonstrating that Black and Hispanic patients experience longer wait times in the Emergency Department than White patients.^34^ Alternatively, this episode may demonstrate how “race-neutral”, clinically appropriate policies of triaging higher over lower acuity cases, or of established protocol for the dental issue the patient was experiencing, may still be interpreted as racist by Black patients or minoritized hospital staff members who have experienced inequities. In this way, a system reliant on adherence to policies and efficiency may overlook a critical component of patient-centered care: understanding patients as individuals who may have had experiences with racist institutions.

### Implications for Practice

Our results suggest that institutional interventions to promote care equity for Black patients should invest in non-clinician staff members, not only in clinicians. The racist experiences respondents shared regarding hiring and division of labor (Q4–8) suggest that institutions should investigate whether these processes are truly equitable. Institutions should examine how increasing wages and ameliorating workload for non-clinician staff members could have a direct impact on equitable patient care. Respondents shared repeatedly how, when workplace conditions were difficult, whether due to inadequate staffing levels or physically demanding work without reprieve, frustration was more likely to erupt between staff members and patients. Some perceived that this disproportionately affected Black patients. Our results indicate that broader interventions, such as increasing staffing levels, ensuring sufficient work breaks, and monitoring fair division of labor by supervisors, could improve employees’ ability to provide high-quality care. Respondents also noted that patients may feel more comfortable with a trustworthy care team, such that staff members with shared identities may be better able to communicate, respond to needs, and de-escalate difficult situations (Q34, Q36). This finding is echoed in the literature on the effects of racial concordance in patient-clinician dyads,^35^ though findings regarding the effects of racial concordance on health outcomes are inconclusive.^36^ To address this, institutions could create educational tools such as dedicated training specifically for hospital staff members to foster empathy and communication skills across racial groups.

Finally, these interviews help us understand how institutional values of diversity and equity are translated into practice. Some respondents appreciated that the hospital system publicly supported DEI-related values (Q42, Q43). Yet, despite the institution’s support of these values in name, some respondents perceived a lack of investment in non-clinician staff members (Q22–25, Q33). Hospitals often pride themselves on being anchor institutions rooted in their local communities.^37^ To authentically support equity, institutions should invest more in non-clinician employees from these same communities. Excluding these employees from DEI interventions creates incomplete solutions to promoting equity. Respondents were only aware of a few DEI initiatives available to them, such as DEI councils and mandatory online educational modules (Q45–47). Yet the institution offers many more, geared toward clinicians higher up in the medical hierarchy. These include programs at the affiliated School of Medicine, scholarship funds, leadership trainings, recruitment and promotions programs, research equity initiatives, clinical outcome monitoring, policy review committees, and community-oriented programs.^38,39^ We recommend that institutions make concerted efforts to include hospital staff members in DEI initiatives. These initiatives should be situated at the structural level, when possible. Individual-level educational initiatives have limited evidence for impacting organization change^8,9^ and tend not to be action-oriented,^40^ though they may help staff recognize how racism manifests in the hospital and prevent themselves or others from discriminating against patients. Structural interventions have the possibility to improve the policies, material conditions, and practices that shape the work and lives of hospital staff members. Reimagining DEI in this broader sense, we suggest initiatives that address respondents’ experiences of being unable to advocate for themselves or patients due to strict hierarchies, cultural norms that undervalued them as care team members (Q33, Q49), and of demanding job requirements that contribute to poor patient care and staff burnout (Q21–24, Q26–28). Examples of such structural initiatives include flattening hierarchies through shared governance models that give non-clinician staff formal decision-making power in unit operations, patient care protocols, and defining reasonable workload volumes; creating protected time and compensation for staff to participate in DEI initiatives; implementing transparent promotion pathways; examining non-clinician staff pay scales to ensure livable wages; and conducting climate surveys with results disaggregated by job role and concrete action plans to address identified issues.

### Limitations

We acknowledge several limitations. Though we describe how racism may operate in experiences of staff members and their interactions with Black patients, other hospital team members may hold different perspectives. Furthermore, social desirability bias may have affected perspectives shared by respondents. We recognize that experiences at the target institution are not necessarily generalizable to others, given its geographic location, population demographics, and other specific institutional identity features. However, while explicit findings may not be generalizable, certain qualities may translate to other institutions based on shared academic structures and the shared US historical context of structural racism and downstream inequities. Another limitation is the inherent power dynamics between interviewers and staff members, who may have self-censored their responses, though this may have been partially mitigated by having two interviewers with different intersectional identities. Similarly, respondents may have self-selected to participate given the interviewers’ perceived identities and recruitment materials indicating the study’s focus on racism. Thus, this study may have captured an incomplete view of staff members’ perspectives.

## CONCLUSION

This qualitative study of 15 hospital staff members reveals how interpersonal and institutional racism operate to perpetuate inequitable care for Black hospitalized patients. Our findings demonstrate that racial inequities may stem from complex interactions between individual biases, workplace discrimination, inadequate staffing and resources, and systemic underinvestment in non-clinician staff, who are predominantly from minoritized communities. This research centers on the underrepresented voices of hospital staff, who are crucial to patient care yet typically excluded from institutional equity initiatives. Our study expands beyond traditional discussions of health inequities, which often focus on clinician perspectives and clinician-centric interventions, by providing a broader institutional perspective from non-clinician hospital staff. Focusing on their voices illuminates how the institutional factors that relate to their work may also influence hospital environments where racist stereotypes persist and Black patients receive suboptimal care.

Hospitals seeking to authentically promote equity and curtail interpersonal and institutional racism must invest in their staff. Practical recommendations include ensuring adequate staffing and manageable workloads; providing living wages and improved working conditions for non-clinician staff; implementing comprehensive, structurally based DEI interventions accessible to staff members; improving mechanisms for staff to report discrimination and advocate for patients and colleagues; and investigating hiring and task allocation practices to eliminate discrimination.

Future research should investigate the effectiveness of interventions targeting institutional practices and non-clinician staff empowerment on care quality and equity outcomes. Studies examining how changes in staffing ratios and wage structures affect staff experiences and patient care could provide evidence for redesigning institutional guidelines. Additionally, research exploring patient perspectives on interactions with staff, focusing on how institutional factors influence these relationships, would complement our findings and inform effective interventions.

## Supplementary Information

Below is the link to the electronic supplementary material.

ESM1 (DOCX 32.0 KB)
