# Supplementary material for: Healthcare Staff Perspectives and Experiences of Racism’s Impact on Care for Hospitalized Black Adults: A Qualitative Study
Source: J Gen Intern Med. 2026 Jan 29;41(12):3370–82. doi: 10.1007/s11606-026-10174-3 (PMC13569782; doi:10.1007/s11606-026-10174-3)
Supplement: Supplementary file 1 [file 11606_2026_10174_MOESM1_ESM.docx]

**Appendix A: Interview guide**

Hello and thank you for taking the time to talk to me. My name is *** and I’m a research assistant on Dr. Liz Dzeng’s research team. We’re investigating racism and equity in the hospital for older Black adult patients.

The purpose of today’s interview is to hear your observations and experiences regarding the medical care of older Black adult patients. We are interested in hearing your experiences for our research study and are not sharing specific information with anybody at [ACADEMIC HOSPITAL]. In other words, nobody will get in ‘trouble’ for anything you share and none of this information will be shared with your supervisors or coworkers. This interview will be audiotaped and transcribed, but everything will be made anonymous, so no one will be able to tie the interview data to you.

We understand it can be difficult to discuss this topic and related experiences. If you would like me to stop the audio-recorder or interview at any time, please let me know. You don’t have to answer any specific question if it makes you uncomfortable.

Do you have any questions before we begin?

**Demographics**

1. Age: _______

2. Gender: ________

3. Do you identify as: [ ] Hispanic [ ] Non-Hispanic

4. Which race do you identify with: [ ] White [ ] Black [ ] Asian, Asian-American, or Pacific Islander [ ] Native American [ ] Multiethnic [ ] Other. Please describe: ___ [ ] Decline to respond

5. How long have you worked as a *** at [ACADEMIC HOSPITAL]? ___________________

6. Which clinical settings have you worked at?

**Introduction to this study**

We are interested in your experience as a valued member of [ACADEMIC HOSPITAL]’s hospital staff. In your unique role, you have probably had many interactions with patients and their loved ones. This study focuses on Black adult patients at [ACADEMIC HOSPITAL]. You may also have seen patterns of how racial differences impact care that older Black patients receive. The following questions address your personal perspectives and observations regarding these topics.

**Introductory questions regarding inequities**

1) **Stem: Do you think that older Black patients are treated differently at [ACADEMIC HOSPITAL] compared to older patients who are not Black?**
   a) Probes – Staff member’s impression of patient care
      i) In what ways did you see Black patients treated differently?
         (1) Can you give any examples?
             (a) Who was treating the patient differently?
             (b) What other reasons contributed to them treating the patient differently?
             (c) Are there any policies or regulations at [ACADEMIC HOSPITAL] that contributed to patients being treated differently?
      ii) *If the respondent feels older Black patients are not treated differently:* What are some reasons that older Black patients are not treated differently at [ACADEMIC HOSPITAL]?
          (1) Are there any policies or regulations at [ACADEMIC HOSPITAL] that contribute to patients being treated the same no matter their race?
          (2) How have you seen hospital staff make an effort to treat patients equally no matter their race?
   b) Probes – Specific impressions regarding serious illness and EOL care. *Note: respondents might not have experience with the following. If so, please skip to part ii.*
      i) Have you observed any instances where older Black patients were treated differently while:
         (1) Being treated for advanced illnesses such as end-stage cancer, heart failure, liver failure, etc.?
         (2) In the ICU?
         (3) On life support machines (such as a ventilator/breathing machine)?
         (4) Undergoing discussions about hospice and/or palliative care?
         (5) On comfort care or hospice?
         (6) Being given pain medications?
         (7) Can you give any examples?
             (a) Who was treating the patient differently?
             (b) What other reasons contributed to them treating the patient differently?
             (c) Are there any policies or regulations at [ACADEMIC HOSPITAL] that contributed to patients being treated differently?

**Transition into next section**

Racism can be one of the reasons why racial inequities exist. The next section will ask about your perspectives on racism.

**Introductory questions regarding racism**

1) **Stem: Do you think that racism in the United States is a problem?**
   a. Probe – Why or why not?
2) **Stem: Do you think that racism against Black patients in healthcare is a problem?**
   a. Probe – Why or why not?
3) **Stem: Have you experienced racism in your life?**
   a. Probe – Have you experienced racism outside of your job?
      i. Can you tell me about that experience? *(from whom, situation, setting)*
         1. How did you respond to this experience?
   b. Probe – Have you experienced racism at [ACADEMIC HOSPITAL]?
      i. Can you give any examples? *(from whom, situation, setting)*
         1. How did you respond to this experience?
         2. Did you report this?
         3. Are there any policies at [ACADEMIC HOSPITAL] that made this experience worse?
         4. Are there any policies at [ACADEMIC HOSPITAL] that helped you get through this experience?
   c. Probe – Have you noticed racial tensions between [*PCAs/transporters/clerks/custodians*] and other groups of staff, like nurses or doctors?
      i. Can you give any examples?
      ii. What are some reasons for these racial tensions?
   d. Probe – How do those experiences impact how you approach your job?
      i. How do those experiences affect how well you feel you can do your job?
      ii. How do those experiences affect how you take care of patients?
      iii. Can you give any examples?

**Witnessing anti-Black racism**

1) **Stem: Have you witnessed anti-Black racism by clinicians or hospital staff in serious illness or end-of-life care at [ACADEMIC HOSPITAL]?**
   a) Probes - Racism by clinicians and hospital staff
      i) Have you observed any instances where [ACADEMIC HOSPITAL] clinicians or staff:
         (1) Expressed racist stereotypes or beliefs about Black patients?
             (a) E.g. beliefs in biological differences based on race
         (2) Treated patients differently due to race (e.g., in clinical care or in conflict situations)?
         (3) Treated patients’ family members or loved ones differently due to their race?
         (4) *If interviewer wants to probe further:* Neglected Black patients?
         (5) *If interviewer wants to probe further:* Disrespected Black patients?
      ii) How did observing those situations make you feel?
      iii) How, if at all, did those experiences impact you afterward?
      iv) Did any of these instances happen to older Black adult patients with serious illnesses (for example, in the ICU, on life support, with advanced cancer)?
          (1) Can you give any examples?
2) **Stem: Do you think that older Black patients tend to have security called on them more often than patients who are not Black?**
   a) If yes:
      i) Why do you think this is the case?
         (1) Do you think staff members have certain stereotypes of older Black patients that influences when they call security?
             (a) Can you share any examples?
      ii) Can you share a time when security was called on an older Black patient?
          (1) How did this make you feel?
          (2) How do you think this should have been handled differently?
   b) How have you noticed security interacting with older Black patients/or Black patients in general?
      i) Do you feel this is different than how they interact with patients of other races?
      ii) If yes:
          (1) Please describe some of these differences
          (2) Why do you think this is the case?
3) **Stem: Have you witnessed older Black patients being restrained/put in restraints?**
   a) If yes:
      i) Can you describe what happened?
         (1) Do you think staff members have certain stereotypes of older Black patients that influences when they use restraints?
             (a) Can you share any examples?
      ii) Have you witnessed non-Black patients being put in restraints?
          (1) If yes: Have you noticed differences in the way restraints are used for Black patients compared to non-Black patients?
      iii) Can you think of a time when restraints were placed on an older Black patient unnecessarily? If so, what happened?
           (1) How did this make you feel?
           (2) How do you think this should have been handled differently?
4) **Stem: How do you think older Black patients have felt about the care they received at [ACADEMIC HOSPITAL]?**
   a) Probes - Patients’ subjective experiences of their care
      i) Can you tell me about a time when a patient expressed dissatisfaction about the quality of care they received?
      ii) Can you tell me about a time when a patient expressed distrust of hospital staff members or clinicians?
      iii) Can you describe a time when patients were frustrated or dissatisfied with one of [ACADEMIC HOSPITAL]’s policies or procedures?
      iv) What other feelings did the patient express regarding their care?
      v) What other feelings did any friends, family, or other caregivers express regarding patients’ care?
   b) What factors do you think contributed to the patient/friends/family feeling this way?
      i) How do you think these factors could be improved?
5) **Stem: Have you noticed anything about your job or your coworkers’ jobs that makes it difficult to treat all patients equally?**
   a) Probes – Institutional factors contributing to inequity
      i) How do you feel that staffing levels impact staff members’ ability to treat all patients equally?
      ii) How do you feel that wage or salary levels impact staff members’ ability to treat all patients equally?
      iii) How do you feel that stress levels impact staff members’ ability to treat all patients equally?
      iv) Do you think there are certain units in the hospital in which it is easier to treat patients equally? *E.g. the Emergency Department, ICU*
          (1) Can you give any examples?
6) **Stem: Have you had experiences with people at [ACADEMIC HOSPITAL] speaking out against racism?**
   a) Probes – Standing up against racism
      i) Have you seen people at work standing up against racism?
         (1) Can you give any examples?
         (2) What went well?
         (3) What didn’t go well?
         (4) What do you think could have been done differently?
      ii) Has there been a time at work when you’ve stood up against racism?
          (1) If yes:
              (a) Could you describe what happened?
              (b) How did you feel after speaking up?
              (c) Did you feel supported after speaking up?
          (2) If no:
              (a) What prevented you from speaking up?
      iii) Do you feel that [ACADEMIC HOSPITAL] supports staff to stand up against racism?
           (1) If yes:
               (a) What are some ways they support staff to stand up against racism?
           (2) If no:
               (a) What aspects about [ACADEMIC HOSPITAL] make it difficult for staff to stand up against racism?
               (b) What do you think could be done differently to support staff standing up against racism?
   b) Probes – Goals for change
      i) If you could make changes at work to combat racism, what would they be?
      ii) Who do you think needs to be responsible for those changes?
      iii) What do you think is standing in the way of those changes?
7) **Stem: Have you had any training at your job about diversity, equity and inclusion?**
   a) If yes:
      i) Can you describe what that training consisted of?
      ii) How did you feel about these trainings?
      iii) How did it change the way you approach your job?
   b) If no:
      i) Do you wish you had more training in this area?
      ii) What would you recommend including in these trainings?
      iii) Have you heard of other staff members getting this type of training?

**Checklist of scenarios *(if time permits):***

- Have you ever noticed disparaging or unkind comments made about an older Black patient outside of the patient’s room?
  - Please describe what happened
  - How did this make you feel?
  - How do you think this should have been handled differently?
- Have you ever noticed Adult Protective Services being involved in the care of older Black patients?
  - Please describe what happened
  - How did this make you feel?
  - How did this impact your ability to do your work?
  - How do you think this should have been handled differently?
- Have you ever noticed risk management or patient relations being involved in the care of older adult patients?
  - Please describe what happened
  - How did this make you feel?
  - How did this impact your ability to do your work?
  - How do you think this should have been handled differently?
- Do you have access to the EMR?
  - If yes:
    - Can you describe instances where race was mentioned in the note?
    - Can you describe instances where patient behavior contracts were drawn up for patients?
    - Can you describe instances where flags were placed in a patient’s chart for any reason?
      - What did the flag say?
      - What circumstances led to the flag getting placed?
      - What impact did the flag have on the patient’s care?

**Approaches to probing racist statements**

- How does *** affect the way you interact with patients?
- What makes you think/feel that way?
- Have any experiences shaped how you think about this topic?

**Wrap-up**

Is there anything I haven’t asked you about that you’d like to share?

That brings us to the end of the interview. Thank you again for sharing your time with us.

**Appendix B: Standards for Reporting Qualitative Research (SRQR) Checklist**

| **Title and abstract** | | **Page/line no(s).** |
| --- | --- | --- |
|  | **Title** - Concise description of the nature and topic of the study Identifying the study as qualitative or indicating the approach (e.g., ethnography, grounded theory) or data collection methods (e.g., interview, focus group) is recommended | 1/1-2 |
|  | **Abstract** - Summary of key elements of the study using the abstract format of the intended publication; typically includes background, purpose, methods, results, and conclusions | 2/1 – 3/9 |
|  |  |  |
| **Introduction** | |  |
|  | **Problem formulation** - Description and significance of the problem/phenomenon studied; review of relevant theory and empirical work; problem statement | 4/1 – 5/8 |
|  | **Purpose or research questio**n - Purpose of the study and specific objectives or questions | 5/9-18 |
|  |  |  |
| **Methods** | |  |
|  | **Qualitative approach and research paradigm** - Qualitative approach (e.g., ethnography, grounded theory, case study, phenomenology, narrative research) and guiding theory if appropriate; identifying the research paradigm (e.g., postpositivist, constructivist/ interpretivist) is also recommended; rationale | 5/20 – 6/11 |
|  | **Researcher characteristics and reflexivity** - Researchers’ characteristics that may influence the research, including personal attributes, qualifications/experience, relationship with participants, assumptions, and/or presuppositions; potential or actual interaction between researchers’ characteristics and the research questions, approach, methods, results, and/or transferability | 6/18 – 7-5; 6/18-19 |
|  | **Context** - Setting/site and salient contextual factors; rationale | 5/21-22 |
|  | **Sampling strategy** - How and why research participants, documents, or events were selected; criteria for deciding when no further sampling was necessary (e.g., sampling saturation); rationale | 6/13-17 |
|  | **Ethical issues pertaining to human subjects** - Documentation of approval by an appropriate ethics review board and participant consent, or explanation for lack thereof; other confidentiality and data security issues | 6/2-3 |
|  | **Data collection methods** - Types of data collected; details of data collection procedures including (as appropriate) start and stop dates of data collection and analysis, iterative process, triangulation of sources/methods, and modification of procedures in response to evolving study findings; rationale | 7/6-18 |
|  | **Data collection instruments and technologies** - Description of instruments (e.g., interview guides, questionnaires) and devices (e.g., audio recorders) used for data collection; if/how the instrument(s) changed over the course of the study | 7/8-17 |
|  | **Units of study** - Number and relevant characteristics of participants, documents, or events included in the study; level of participation (could be reported in results) | 8/14-17 |
|  | **Data processing** - Methods for processing data prior to and during analysis, including transcription, data entry, data management and security, verification of data integrity, data coding, and anonymization/de-identification of excerpts | 7/14 – 8-12 |
|  | **Data analysis** - Process by which inferences, themes, etc., were identified and developed, including the researchers involved in data analysis; usually references a specific paradigm or approach; rationale | 7/14 – 8-12 |
|  | **Techniques to enhance trustworthiness** - Techniques to enhance trustworthiness and credibility of data analysis (e.g., member checking, audit trail, triangulation); rationale | 7/14 – 8-12 |
|  |  |  |
| **Results/findings** | |  |
|  | **Synthesis and interpretation** - Main findings (e.g., interpretations, inferences, and themes); might include development of a theory or model, or integration with prior research or theory | 8/18 – 14/11 |
|  | **Links to empirical data** - Evidence (e.g., quotes, field notes, text excerpts, photographs) to substantiate analytic findings | 29 - 35 |
|  |  |  |
| **Discussion** | |  |
|  | **Integration with prior work, implications, transferability, and contribution(s) to the field -** Short summary of main findings; explanation of how findings and conclusions connect to, support, elaborate on, or challenge conclusions of earlier scholarship; discussion of scope of application/generalizability; identification of unique contribution(s) to scholarship in a discipline or field | 14/12 – 20/12 |
|  | **Limitations** - Trustworthiness and limitations of findings | 20/14-23 |
|  |  |  |
| **Other** | |  |
|  | **Conflicts of interest** - Potential sources of influence or perceived influence on study conduct and conclusions; how these were managed | 24/2 |
|  | **Funding** - Sources of funding and other support; role of funders in data collection, interpretation, and reporting | 23/8 |
|  |  |  |
|  | **Reference:** |  |
|  | O'Brien BC, Harris IB, Beckman TJ, Reed DA, Cook DA. **Standards for reporting qualitative research: a synthesis of recommendations.** *Academic Medicine*, Vol. 89, No. 9 / Sept 2014  DOI: 10.1097/ACM.0000000000000388 | |
